# Population prevalence of high-risk pharmacogenetic configurations in a healthy older Australian reference cohort

**DOI:** 10.3389/fphar.2026.1881869

**Published:** 2026-09-17

**Authors:** Kosar Hooshmand, Stephen Hughes, Sophie L. Stocker, Andrew A. Somogyi, Mandy L. Ballinger, David M. Thomas

**Affiliations:** 1 Centre for Molecular Oncology, School of Biomedical Sciences, The University of New South Wales, Sydney, NSW, Australia; 2 Sydney Pharmacy School, Faculty of Medicine and Health, The University of Sydney, Sydney, NSW, Australia; 3 Faculty of Medicine, St. Vincent’s Clinical Campus, University of New South Wales, Sydney, NSW, Australia; 4 Department of Clinical Pharmacology and Toxicology, St. Vincent’s Hospital, Sydney, NSW, Australia; 5 Discipline of Pharmacology, College of Health, Adelaide University, Adelaide, SA, Australia; 6 Omico, the Australian Genomic Cancer Medicine Centre, Kensington, NSW, Australia

**Keywords:** ClinPGx/PharmGKB, CPIC, high-risk pharmacogenetic variants, opioid response, pharmacogenetics, whole-genome sequencing

## Abstract

Recent advances in pharmacogenomic guidelines, regulatory labelling, and clinical implementation are supporting broader adoption of pharmacogenetic testing. We analysed whole-genome sequencing data from 3,205 participants in the Medical Genome Reference Bank, a healthy older Australian reference cohort predominantly of European ancestry, to characterise established and novel pharmacogenetic variation. Overall, 1,303 participants (40.7%) carried at least one confirmed homozygous ClinPGx/PharmGKB Level 1A high-risk configuration. A further 399 participants (12.4%) carried at least one potential-trans, phase-unresolved configuration. After accounting for overlap, 1,566 participants (48.9%) had either a confirmed homozygous or potential-trans, phase-unresolved configuration, representing an upper-bound estimate of potential biallelic involvement rather than confirmed biallelic prevalence. Confirmed homozygous configurations occurred in two or more high-risk pharmacogenes in 255 participants (8.0%) and in three or more genes in 22 participants (0.7%); the corresponding upper-bound estimates were 377 (11.8%) and 45 (1.4%), respectively. None of 409 candidate potential-trans participant–gene configurations could be directly resolved as cis or trans from available phase information. A total of 140 novel predicted loss-of-function participant–gene category records were identified across the high-risk pharmacogene set. Within *CYP2D6*, 277 participants (8.6%) carried confirmed homozygous Level 1A configurations, although these rsID-defined findings do not represent a complete estimate of *CYP2D6* metaboliser-phenotype prevalence. Exploratory structural modelling of selected *OPRM1* missense variants identified variation in predicted fentanyl docking scores and receptor–ligand contact geometry, although these findings require experimental validation. These results demonstrate the value of population-scale whole-genome sequencing for pharmacogenomic discovery while highlighting the importance of distinguishing confirmed homozygous findings from phase-unresolved multi-variant configurations.

## 1 Introduction

Prescribing frequently involves a trial-and-error approach (Howes et al., 2022), which may contribute to ineffective treatment and adverse drug reactions (ADRs) (Paul and Potter, 2024). Pharmacogenomics offers a means of reducing these risks by informing drug selection and dosing according to an individual’s genetic profile. Over the past decade, the field has advanced substantially through the development of mature international guidelines from the Clinical Pharmacogenetics Implementation Consortium (CPIC), the Dutch Pharmacogenetics Working Group (DPWG), the Canadian Pharmacogenomics Network for Drug Safety (CPNDS), and the French National Network of Pharmacogenetics (RNPGx); pharmacogenomic labelling updates from the U.S. Food and Drug Administration (FDA) and European Medicines Agency (EMA); implementation studies; and improvements in clinical informatics and decision-support infrastructure (Abdullah-Koolmees et al., 2021). Collectively, these developments have increased the feasibility of clinical implementation while also highlighting the need for additional population-specific and variant-level evidence.

Inherited variation in pharmacogenes encoding drug-metabolising enzymes, transporters, and receptors can influence drug absorption, disposition, efficacy, and toxicity across a broad range of clinical specialties, including oncology, cardiology, psychiatry, infectious diseases, gastroenterology, pain management, and anaesthesia (Pirmohamed, 2023; Jaya Shankar et al., 2022; Auwerx et al., 2022; Katara and Yadav, 2019). Although many pharmacogenomic recommendations are gene specific, clinically relevant prescribing may involve more than one pharmacogene. Examples include solute carrier organic anion transporter family member 1B1 (*SLCO1B1*) and cytochrome P450 family two subfamily C member 9 (*CYP2C9*) in statin prescribing (Cooper-DeHoff et al., 2022), *CYP2C19* and *CYP2D6* in selected psychotropic prescribing contexts (Austin-Zimmerman et al., 2021), thiopurine S-methyltransferase (*TPMT*) and nudix hydrolase 15 (*NUDT15*) in thiopurine therapy (Pratt et al., 2022), and ryanodine receptor 1 (*RYR1*) and calcium voltage-gated channel subunit alpha1 S (*CACNA1S*) in malignant-hyperthermia susceptibility (Fiszer et al., 2015). Characterising such multi-gene patterns may therefore provide a broader view of pharmacogenomic variation relevant to personalised prescribing (Cross et al., 2022).

Population-scale whole-genome sequencing (WGS) provides an opportunity to identify both established pharmacogenetic variants and rare or previously uncharacterised variants that may not be detected by targeted assays. However, many pharmacogenomic interpretations depend on haplotypes, star alleles, diplotypes, metaboliser phenotypes, copy-number states, or complex structural configurations. Variant-centric analyses of short-read WGS data must therefore be interpreted cautiously when haplotype phase, copy-number variation, or complex structural alleles cannot be resolved. This is particularly important for genes such as *CYP2D6* and dihydropyrimidine dehydrogenase (*DPYD*), in which multi-variant haplotypes, structural variation, and cis/trans configuration may affect clinical interpretation. For *CYP2D6* in particular, accurate functional interpretation may additionally require copy-number-aware and hybrid-allele-aware analysis because deletions, duplications, and *CYP2D6*–*CYP2D7* hybrid alleles cannot be comprehensively resolved using a simple rsID-defined variant-centric framework.

Distinguishing homozygous from multi-variant configurations is also essential when estimating potential biallelic pharmacogenomic variation. A homozygous non-reference genotype is phase independent because both copies of the locus carry the same alternate allele. In contrast, two distinct heterozygous variants within the same gene can occur in cis on the same homologue or in trans on opposite homologues. Without direct phase evidence, such configurations cannot be considered confirmed compound heterozygous or confirmed biallelic findings. Analyses based on unphased short-read WGS should therefore report confirmed homozygous findings separately from potential-trans, phase-unresolved configurations and should describe any combined estimate as an upper bound rather than as confirmed biallelic prevalence.

In this study, we analysed WGS data from 3,205 participants in the Medical Genome Reference Bank (MGRB), a healthy older Australian reference cohort enriched for individuals aged 70 years or older and predominantly of European ancestry. This cohort provides a valuable resource for estimating pharmacogenomic variation in healthy older Australians; however, the findings should be interpreted as cohort-specific rather than as directly generalisable estimates for all Australians or other populations. Pharmacogene allele frequencies and haplotype distributions vary across ancestry groups, and the disease-free older cohort design may introduce healthy-survivor and ascertainment biases.

This study had three main aims. First, we characterised known clinically relevant pharmacogenetic variation across a curated 38-gene analytic set and a focused 12-gene high-risk subset defined by ClinPGx/PharmGKB Level 1A evidence and CPIC guideline-based prescribing relevance. For the high-risk analysis, confirmed homozygous configurations were reported separately from within-gene multi-variant configurations. Recurrent multi-variant patterns were reviewed using established PharmVar, CPIC, and ClinPGx allele or haplotype definitions, and available VCF phase fields were audited to determine whether any candidate configurations could be directly resolved as cis or trans. Potential-trans configurations lacking complete participant-level phase evidence were retained as phase unresolved and were included only in an explicitly labelled upper-bound estimate.

Second, we catalogued rare and novel predicted loss-of-function and missense variants to support future pharmacogenomic interpretation. These findings were analysed separately from the primary ClinPGx/PharmGKB Level 1A estimates and were not considered clinically actionable solely on the basis of predicted functional consequence.

Third, we conducted an exploratory opioid-response case study focused on *OPRM1*. AlphaFold-based structural modelling and molecular docking were used to investigate predicted ligand-positioning and docking-score differences associated with selected missense variants (Jumper et al., 2021). These analyses were intended to provide hypothesis-generating structural context and were not used to assign pharmacogenomic phenotypes or clinical actionability.

Together, this approach integrates population-level pharmacogene variation with pathway- and protein-level interpretation while distinguishing rsID-defined variant observations from directly phased haplotypes, diplotypes, and metaboliser phenotypes. An overview of the cohort, data-processing, curation, classification, and analytical workflow is provided in Supplementary Figure S1.

## 2 Materials and methods

### 2.1 Study cohort and whole-genome sequencing data processing

This study analysed WGS data from 3,205 participants in the Medical Genome Reference Bank (MGRB), a previously described healthy older Australian reference cohort (Pinese et al., 2020). Participants included in the present analysis were aged ≥70 years and had no reported history of cancer, cardiovascular, or neurodegenerative disease at cohort ascertainment. The cohort was used here to estimate the prevalence of clinically relevant pharmacogenomic variation in a healthy older reference population.

Individual-level age and sex metadata were available for the analysed WGS dataset and were summarised as available cohort characteristics. The term “Australian” refers to the recruitment and enrolment context of the cohort and should not be interpreted as a proxy for genetic ancestry. Genetic ancestry was assessed using principal-component analysis as previously described for the MGRB cohort (Pinese et al., 2020). The analysed cohort was predominantly of European ancestry.

Whole-genome sequencing was performed at an average depth of 38×. Sequence reads were aligned to the GRCh38 human reference genome, and variant-calling and quality-control were performed using the established MGRB processing pipeline, as previously described (Pinese et al., 2020).

Concurrent medication exposure, prescribing indication, detailed comorbidity data, and prospectively collected drug-response or adverse-event outcomes were not uniformly available for the analysed dataset. Consequently, the primary analysis focused on genotype prevalence and did not assess medication-specific exposure, disease-specific prescribing context, comorbidity-stratified pharmacogenomic risk, or observed treatment outcomes.

### 2.2 Gene selection, pharmacogenetic variant curation, and annotation

The gene-selection strategy was designed to identify pharmacogenes with clinically relevant variant-level evidence while maintaining a transparent and reproducible framework for downstream WGS analysis. Pharmacogenes and associated variants were curated from the CPIC guideline resources (https://cpicpgx.org/guidelines/; release 2022; accessed 2024-08-14) and ClinPGx/PharmGKB clinical association resources (https://www.clinpgx.org/downloads; release 2022; accessed 2024-08-16).

The evidence frameworks used by CPIC and ClinPGx/PharmGKB were treated separately. CPIC assigns clinical actionability levels to gene–drug pairs and provides prescribing recommendations, whereas ClinPGx/PharmGKB assigns evidence levels, including Levels 1A, 1B, 2A, and 2B, to pharmacogenomic variant–drug or gene–drug associations. Accordingly, references to Levels 1A, 1B, 2A, and 2B in this study refer to the ClinPGx/PharmGKB clinical annotation evidence framework. CPIC resources were used to establish prescribing relevance and to support the interpretation of allele function, diplotype-to-phenotype mappings, and clinical recommendations.

The analyses were based on the archived 2022 source releases accessed in August 2024. Because these resources are periodically updated, the curated catalogue reflects the specified archived releases rather than the current live database content.

The starting point for the analysis was not a current top-level CPIC gene–drug pair list or a current top-level ClinPGx pharmacogene list alone. Instead, a curated variant-level pharmacogene catalogue was assembled by integrating PharmGKB/ClinPGx clinical annotations, CPIC guideline-derived information, and PharmVar or star-allele reference annotations where available. The catalogue retained gene symbols, rsIDs, genomic coordinates, REF/ALT alleles, PharmGKB/ClinPGx allele labels, altered alleles, evidence levels, associated chemicals/drugs, reported phenotypes, and ancestry-stratified allele frequencies where these fields were available.

Very important pharmacogenes (VIP) with ClinPGx/PharmGKB clinical annotation Levels 1A, 1B, 2A, or 2B were prioritised, together with Level 1A variants with CPIC guideline-based prescribing relevance. This yielded an initial curated panel of 42 pharmacogenes.

Classical human leukocyte antigen (*HLA*) pharmacogenomic alleles were not included in the downstream rsID-defined analysis. Although *HLA*-A and *HLA*-B contain clinically important ClinPGx/PharmGKB and CPIC pharmacogenomic associations, including *HLA*-B*57:01, *HLA*-B*15:02, *HLA*-B*15:11, *HLA*-B*58:01, and *HLA*-A*31:01, these Level 1A annotations correspond to classical HLA allele or haplotype calls rather than individual rsID-defined SNV or short-indel variants. In the ClinPGx/PharmGKB source records used for curation, these Level 1A HLA allele entries did not have directly usable rsID, CHR/POS, REF, or ALT definitions suitable for direct VCF querying. Accurate detection of these alleles from short-read WGS requires dedicated *HLA* typing or validated *HLA* imputation methods, which were outside the scope of the present variant-centric pipeline.

Although some rsID-level variants within *HLA*-A and *HLA*-B were present in the source variant exports, these entries did not carry Level 1/2 clinical annotation evidence in the curated variant-level dataset and did not correspond to validated classical *HLA* allele calls. Therefore, they were not included as substitutes for clinically actionable *HLA* haplotypes. *HLA*-A and *HLA*-B were excluded from the 38-gene VIP-wide analysis and the 12-gene high-risk rsID-defined participant-level analysis. This exclusion reflects analytic scope rather than lack of clinical importance.

To maintain relevance to general-population prescribing and minimise indication-specific bias, four genes were excluded *a priori* from downstream analysis: cystic fibrosis transmembrane conductance regulator (*CFTR*; scope largely confined to cystic fibrosis modulator therapy), epidermal growth factor receptor (*EGFR*; primarily oncology tyrosine-kinase inhibitors), vitamin K epoxide reductase complex subunit 1 (*VKORC1*; near-universal actionable genotype in this cohort, limiting stratification value), and X-ray repair cross complementing 1 (*XRCC1*; no Level 1A variants observed in our dataset). After these exclusions, 38 pharmacogenes remained for downstream VIP-wide analyses.

Within the 38-gene analytic set, a focused 12-gene high-risk subset was predefined for the primary analysis. In this study, “high-risk” refers to an analytic category defined by three criteria: ClinPGx/PharmGKB Level 1A evidence, high-priority electronic health record or clinical actionability notation in the curated interpretation tables, and CPIC guideline-based prescribing relevance. This definition was not based on cohort allele frequency, drug-class breadth, or predicted functional consequence alone. The 12 high-risk genes were ATP binding cassette subfamily G member 2 (*ABCG2*), CYP2 subfamily B member 6 (*CYP2B6*), *CYP2C9*, *CYP2C19*, *CYP2D6*, CYP3 subfamily A member 5 (*CYP3A5*), *DPYD*, *G6PD*, *NUDT15*, RYR1, *SLCO1B1*, and *TPMT*. Variants in these genes have established relevance to treatment selection, dose optimisation, toxicity avoidance, or other clinically actionable prescribing decisions.

The supplementary tables document the curation and filtering workflow. Supplementary Table S1 contains the full 42-gene starting catalogue of curated pharmacogenetic variants. Supplementary Table S2 provides variant- and allele-level interpretation fields, including allele combinations, activity scores, coded diplotypes/phenotypes, EHR priority notation, and clinical recommendation text. Supplementary Table S3 provides the final ClinPGx/PharmGKB Level 1A subset used for the focused high-risk analysis.

Supplementary Table S3 was generated by merging gene-specific annotation sources where available, including allele-definition tables, diplotype–phenotype tables, and coding-sequence or matched-variant output files linking rsIDs and genomic variant records to curated pharmacogenomic annotations. Allele-definition tables provided allele and function context, diplotype–phenotype tables provided phenotype or activity-score interpretation where defined by the source resource, and coding-sequence or matched-variant outputs linked these annotations to rsID-level variants observed in the WGS dataset.

Activity-score, allele-function, and diplotype–phenotype fields were retained where defined in the source files. These fields were not available or applicable for every variant-level row because activity scores are often defined at the allele, diplotype, or phenotype level rather than as standalone annotations for each individual rsID. Where no source-defined activity score was available for a variant-level row, or where interpretation required diplotype-level, phase-resolved, or copy-number-aware calling, the activity-score field in Supplementary Table S3 was marked as not available, not applicable, or requiring diplotype-level interpretation rather than left blank.

Star-allele nomenclature, allele-function annotations, activity-score fields, and diplotype/phenotype mappings were retained in the supplementary tables for interpretive context only. Definitive star-allele haplotypes, diplotypes, metaboliser phenotypes, and participant-level activity scores were not assigned in the primary analysis.

This limitation is particularly important for *CYP2D6*. The present rsID-defined short-read WGS analysis did not comprehensively resolve *CYP2D6* copy-number variation, whole-gene deletions such as CYP2D6*5, gene duplications, *CYP2D6*–*CYP2D7* hybrid alleles, or other complex structural configurations. Accordingly, definitive *CYP2D6* star alleles, diplotypes, activity scores, and metaboliser phenotypes were not assigned, and the *CYP2D6* findings reported in this study represent only the subset of variation detectable within the present variant-centric analytical framework.

Primary genotype analyses were performed at the individual variant or rsID level, consistent with the ClinPGx/PharmGKB variant-centric evidence framework.

PharmVar and CPIC or ClinPGx star-allele definitions were subsequently used as part of an interpretability review of recurrent within-gene multi-variant configurations. This review was used to identify configurations that were compatible with known cis-associated haplotypes and configurations that could potentially represent variants on opposite homologues. These assignments were treated as interpretive categories rather than definitive phase-resolved diplotypes. Multi-variant configurations without direct phase evidence were therefore classified as likely cis or haplotype-associated, potential trans with phase unresolved, or otherwise unresolved, as described in Section 2.4.

ClinPGx integrates pharmacogenomic resources including PharmGKB, CPIC, the Pharmacogene Variation Consortium (PharmVar), the Pharmacogenomics Clinical Annotation Tool (PharmCAT), FDA, and DPWG resources, providing harmonised haplotype nomenclature and cross-consensus clinical annotations. Custom Python scripts normalised ClinPGx/PharmGKB and CPIC-derived annotations, expanded multi-allelic entries, merged records by gene–allele pair and rsID where available, and retained variant characteristics including evidence level, associated drugs and predicted functional consequences.

SNP annotation was performed in R using biomaRt v.2.58.2 (Durinck et al., 2005), with data processing using dplyr v.1.1.4. Processed datasets were exported to VCF format for integration with the MGRB cohort and downstream genomics workflows, then compressed and indexed using bcftools v.1.21 (Danecek and McCarthy, 2017), and htslib v.1.16 (Bonfield et al., 2021) for scalable, high-throughput querying.

### 2.3 Statistical analysis of VIP and high-risk variants

Analyses were conducted at two levels: a VIP-wide analysis comprising curated variants with ClinPGx/PharmGKB clinical annotation Levels 1A–2B across the 38-gene analytic set, and a focused high-risk analysis restricted to variants with ClinPGx/PharmGKB Level 1A evidence, high-priority clinical actionability notation, and CPIC guideline-based prescribing relevance.

For each curated variant, genotypes were extracted from the MGRB whole-genome sequencing dataset and matched to harmonised rsID, genomic-coordinate, and allele definitions derived from ClinPGx/PharmGKB, CPIC, and PharmVar resources. ClinPGx/PharmGKB allele annotations and CPIC-derived allele-function, diplotype-to-phenotype, and prescribing information were used as interpretive reference material. Definitive star-allele haplotypes, diplotypes, metaboliser phenotypes, participant-level activity scores, and copy-number-aware pharmacogene calls were not assigned from the sequencing data. Primary genotype analyses were therefore conducted at the individual variant or rsID level, as documented in Supplementary Tables S2 and S3.

Custom Python pipelines processed the MGRB VCFs and curated ClinPGx/PharmGKB and CPIC-derived annotation files in parallel batches, with consistent treatment of multiallelic sites. For each variant, alternate-allele counts, genotype counts, unique carrier counts, and cohort allele frequencies were calculated. Where variant allele fraction was reported, it referred to the proportion of sequencing reads supporting the alternate allele within an individual sample. Population-level summaries were reported as allele frequency and were not described as variant allele fraction.

VIP-wide genotype distributions and allele frequencies across the 38-gene analytic set are reported in Supplementary Table S4. Results for the focused 12-gene high-risk analysis are reported in Supplementary Table S5, including heterozygous genotypes, homozygous non-reference genotypes, and within-gene multi-variant configurations. Participant-level homozygous and multi-variant configurations were classified according to the interpretability, phase-audit, and non-redundant hierarchical framework described in Section 2.4. The supporting haplotype and phase-interpretability review is reported in Supplementary Table S6. Final mutually exclusive participant-level and participant-gene classifications, including phase-status categories, are provided in Supplementary Table S7.

To assess the reliability of variant detection across the focused high-risk pharmacogene set, gene-level sequencing coverage was summarised for the 12 high-risk genes in all 3,205 analysed MGRB samples. For each gene, per-sample mean depth, median depth, target size, and the proportion of target bases covered at thresholds of at least 10×, 20×, and 30× were calculated across the analysed genomic intervals. Mean gene-level sequencing depth ranged from 31.05× for G6PD to 43.29× for CYP2D6. Coverage at 20× or greater was high for most high-risk pharmacogenes, although G6PD showed comparatively lower coverage, with a mean of 78.99% of target bases covered at this threshold. Gene-level coverage summaries are provided in Supplementary Table S8.

Functional annotation was performed using the Ensembl Variant Effect Predictor version 111.0 (McLaren et al., 2016). Reported fields included VEP consequence and impact terms, protein-level HGVS annotations, ClinVar clinical significance, and SIFT and PolyPhen predictions where available (Flanagan et al., 2010). These annotations were used descriptively and to provide variant-prioritisation context. They were not used to define inclusion in the primary ClinPGx/PharmGKB Level 1A high-risk set, which was determined from curated ClinPGx/PharmGKB Level 1A evidence, high-priority electronic health record or clinical actionability notation, and CPIC guideline-based prescribing relevance. Where *in silico* prediction tools disagreed, no individual predictor was treated as definitive, and discordant results were retained and interpreted conservatively.

Exploratory per-variant frequency comparisons were performed between the observed MGRB carrier frequency and an expected reference carrier frequency derived from the ClinPGx/PharmGKB “All populations” frequency field where an appropriate reference value was available. These analyses were not used for inter-gene comparisons, clinical-outcome testing, or definition of the primary high-risk variant set.

MGRB carriers were defined as participants with either a heterozygous or homozygous non-reference genotype at the variant under investigation. Where the ClinPGx/PharmGKB “All populations” field represented alternate-allele frequency, denoted by (p), the expected proportion of participants carrying at least one alternate allele was estimated under Hardy–Weinberg equilibrium as (2p (1−p) + p^2^), equivalent to (2p−p^2^). The expected reference carrier count was calculated by multiplying this estimated carrier proportion by the cohort size of 3,205 and rounding to the nearest integer. Where a source field explicitly represented carrier frequency rather than allele frequency, that carrier frequency was multiplied directly by 3,205. The frequency-field definition used for each comparison was verified before calculation.

For both the observed MGRB group and the expected reference group, non-carrier counts were calculated as 3,205 minus the corresponding carrier count. Fisher’s exact test was then applied to a 2 × 2 table comparing carrier and non-carrier counts between the MGRB cohort and the expected reference population.

For each comparison, odds ratios, 95% confidence intervals, nominal two-sided p-values, Benjamini–Hochberg false-discovery-rate-adjusted p-values, and Bonferroni-adjusted p-values were reported. Because multiple exploratory variant-level comparisons were conducted, adjusted p-values were included to aid interpretation, while nominal p-values were retained for transparency. These analyses were interpreted as descriptive frequency comparisons and not as evidence of pharmacogenomic clinical association.

Per-variant VEP annotations, SIFT and PolyPhen predictions, ClinVar clinical significance fields, MGRB carrier counts, reference-frequency definitions, expected carrier counts, odds ratios, 95% confidence intervals, nominal p-values, false-discovery-rate-adjusted p-values, Bonferroni-adjusted p-values, and comparison descriptions are provided in Supplementary Table S9.

### 2.4 Definition and classification of rsID-defined homozygous and multi-variant configurations

Participant-level analyses were performed using a variant-centric framework at the rsID level, consistent with the ClinPGx/PharmGKB evidence framework used for variant annotation. The primary analysis did not assign definitive star-allele haplotypes, diplotypes, metaboliser phenotypes, or copy-number-aware pharmacogene calls. This limitation is particularly relevant to CYP2D6, for which accurate functional interpretation may depend on copy-number variation, gene deletion or duplication, hybrid CYP2D6–CYP2D7 alleles, and phase-resolved haplotype assignment.

For each participant and gene, qualifying ClinPGx/PharmGKB Level 1A variants were aggregated and assigned to mutually exclusive rsID-level configuration categories. A confirmed homozygous configuration was defined as a homozygous non-reference genotype for a qualifying variant at a single locus. Because both copies of the alternate allele are present at the same locus, these configurations were considered phase independent.

A within-gene multi-variant configuration was defined as the presence of two or more distinct qualifying non-reference variants, represented by different rsIDs, in the same gene and participant. A single heterozygous configuration was defined as one qualifying heterozygous non-reference variant in a gene without another qualifying variant in that gene. Male G6PD hemizygous calls were classified separately and were not described as homozygous or biallelic.

Within-gene multi-variant configurations were not automatically classified as compound heterozygous, trans, or confirmed biallelic. Distinct variants observed in the same participant may occur in cis on the same chromosome or in trans on opposite homologues. Consequently, multi-variant configurations required additional interpretive assessment and, where possible, direct evaluation of available phase information.

#### 2.4.1 Haplotype and star-allele interpretability review

The component rsIDs of recurrent multi-variant configurations were compared with established allele and haplotype definitions from PharmVar, CPIC, and ClinPGx resources where these definitions were available. Population-level linkage disequilibrium and co-occurrence information was considered as supporting context but was not treated as direct evidence of participant-level phase.

Configurations corresponding to recognised same-haplotype structures were classified as likely cis or haplotype-associated. For example, the *CYP2B6* combination rs3745274 and rs2279343 was considered compatible with a CYP2B6*6*-*associated haplotype structure*.* Other recognised combinations involving DPYD, SLCO1B1*15, TPMT*3A, or *CYP2C19*-associated haplotypes were similarly classified as likely cis or haplotype-associated where supported by established allele definitions.

Configurations containing markers associated with distinct or potentially independent allele structures were classified as potential trans, phase unresolved when the variants could plausibly occur on opposite homologues but direct participant-level phase evidence was unavailable or incomplete. For example, a configuration comprising the likely cis-associated *CYP2B6* rs3745274–rs2279343 component together with rs3211371 was considered potentially compatible with variants on opposite homologues. However, it was not classified as confirmed trans without direct phase evidence.

Multi-variant configurations that could not be confidently aligned with a recognised cis-associated haplotype or a plausible potential-trans structure were retained as unresolved multi-variant configurations.

These interpretive categories were not considered definitive haplotype or diplotype assignments. In particular, the category “potential trans, phase unresolved” denotes a configuration that may be compatible with compound heterozygosity but for which trans orientation was not demonstrated.

#### 2.4.2 Direct audit of available VCF phase information

Available genotype-level phase fields in the chromosome-specific MGRB VCFs were examined for configurations classified as potential trans, phase unresolved. The evaluated fields included genotype (GT), physical phasing genotype (PGT), physical phasing identifier (PID), and phase set (PS). Sequencing depth (DP) and genotype quality (GQ) were retained as supporting genotype-quality fields.

A multi-variant configuration was considered directly phase resolved only when all relevant heterozygous variants in that participant and gene had usable phased genotypes and could be assigned to the same physical phase block using a shared, non-missing PID or PS value. Within a shared phase block, alternate alleles occurring on the same haplotype would support a directly phased cis configuration, whereas alternate alleles occurring on opposite haplotypes would support a directly phased trans configuration.

A pipe-delimited genotype alone was not considered sufficient evidence of long-range cis or trans phase when the contributing variants lacked a shared physical phase-block identifier. Similarly, configurations containing only one phased variant, variants assigned to different phase blocks, or missing or incomplete PGT, PID, or PS information remained phase unresolved.

Direct confirmation of unresolved configurations would require a validated phase-resolved method, such as read-backed phasing with sufficient spanning information, long-read sequencing, family-based segregation, validated pharmacogene haplotyping, or another appropriately validated approach. Population-level co-occurrence resources were not treated as substitutes for participant-level phasing.

#### 2.4.3 Final participant–gene classification

A hierarchical assignment was applied so that each participant contributed only one final configuration class per gene. The hierarchy was:

1. confirmed homozygous configuration;
2. Directly phased trans configuration, where demonstrated;
3. Potential trans, phase unresolved;
4. Likely cis or haplotype-associated multi-variant configuration;
5. Unresolved multi-variant configuration; and
6. Single heterozygous configuration.

Male *G6PD* hemizygous calls and variants classified as novel candidate pharmacogenetic findings were retained as separate categories where applicable.

Confirmed homozygous configurations were included in the confirmed homozygous prevalence estimate. Multi-variant configurations were included as confirmed compound heterozygous or confirmed biallelic only when direct trans phase was demonstrated. Potential-trans configurations without complete shared-block phase evidence were reported separately and were used only in an explicitly labelled upper-bound estimate of potential biallelic prevalence. Likely cis or haplotype-associated and otherwise unresolved multi-variant configurations were not included in either the confirmed homozygous prevalence estimate or the upper-bound category. The final mutually exclusive participant–gene classifications, participant-level prevalence categories, and phase-audit status are reported in Supplementary Table S7.

### 2.5 Identification and analysis of novel candidate pharmacogenetic variants

A variant was classified as a novel candidate pharmacogenetic variant for this study when it met all of the following criteria: it was absent from the curated ClinPGx/PharmGKB and CPIC pharmacogenomic variant catalogue; it was not represented in established PharmVar or other curated star-allele definitions for the relevant gene; and it was either absent from gnomAD version 3.1.2 or had a reported global alternate-allele frequency (AF) below 1 × 10^−4^ (Chen et al., 2024).

ClinVar annotations from the 2024 release were retained to provide clinical interpretation context (Landrum et al., 2018). Variants already classified as pathogenic or likely pathogenic for the same gene–variant context were not treated as novel uncharacterised pharmacogenetic findings. Variants classified as variants of uncertain significance, conflicting interpretations, or without a ClinVar assertion were retained as uncharacterised candidates and reported with their corresponding ClinVar status. Variants classified as benign or likely benign were retained in broader annotation outputs but were not interpreted as clinically actionable novel pharmacogenetic variants on the basis of ClinVar evidence.

The primary novel-variant endpoint focused on predicted loss-of-function variants with VEP consequences of stop_gained or frameshift_variant. These variants were prioritised because protein-truncating consequences may substantially alter gene function and are commonly prioritised in clinical variant-interpretation frameworks, including the joint guidance of the American College of Medical Genetics and Genomics (ACMG) and the Association for Molecular Pathology (Richards et al., 2015). Nevertheless, predicted loss of function alone was not considered evidence of pharmacogenomic actionability. Novel predicted loss-of-function variants were therefore described as putatively impactful candidates requiring gene-specific functional and clinical interpretation.

Missense variants were evaluated separately in a transcript-aware protein-level catalogue using VEP protein HGVS annotations. ClinVar clinical significance fields were used to distinguish pathogenic or likely pathogenic, benign or likely benign, uncertain, conflicting, and uncharacterised findings. Only uncharacterised missense variants with an interpretable amino-acid substitution were considered for exploratory structural modelling. Structural modelling was used as supportive exploratory evidence and not as a basis for assigning clinical pathogenicity or pharmacogenomic actionability.

Variant normalisation, genotype quality control, and cohort-level aggregation followed the procedures described in Sections 2.2–2.4. Cohort VCFs were queried to generate participant–gene records containing variant consequence and genotype information. Each participant–gene combination contributed only once to participant-level summaries through the non-redundant classification hierarchy described in Section 2.4.

Homozygous novel predicted loss-of-function variants were considered phase independent. Participants carrying two or more distinct novel candidate variants within the same gene were recorded as having a novel multi-variant configuration. Such configurations were not considered confirmed compound heterozygous or confirmed biallelic without direct phase evidence. Where established haplotype information was available, the variants were reviewed using the same interpretability principles applied to known high-risk variants. Where sufficient haplotype or direct phase information was unavailable, they were retained as novel multi-variant observations requiring further validation.

The final outputs included a curated catalogue of novel predicted loss-of-function variants, variant-level carrier counts stratified by genotype, and participant–gene summaries of novel predicted loss-of-function findings across the high-risk pharmacogene set. Heterozygous carriers were also summarised separately to describe the broader burden of potentially relevant variation. Novel heterozygous, confirmed homozygous, and unresolved multi-variant findings were reported as distinct categories and were not combined into a single confirmed biallelic estimate. Detailed variant-level and participant–gene results for novel candidate pharmacogenetic variants are provided in Supplementary Table S10.

### 2.6 Molecular docking analysis and structural visualisation of the OPRM1 missense variants

To explore the potential structural effects of selected missense variants in *OPRM1*, wild-type and variant-specific models of the μ-opioid receptor were generated using ColabFold v.1.5.5, an AlphaFold2-based protein-structure prediction implementation (Mirdita et al., 2022). The reference OPRM1 protein sequence corresponding to the GRCh38 assembly was obtained from Ensembl. Variant-specific protein sequences were generated by introducing the relevant amino-acid substitutions into the reference sequence. Where coding-sequence translation was required, nucleotide sequences were translated using ExPASy v.3.0 (Gasteiger et al., 2003).

Model confidence was assessed using AlphaFold-derived predicted local distance difference test (pLDDT) scores and predicted aligned error (PAE) matrices. Structural interpretation was restricted to receptor regions with acceptable local confidence. Residues and regions with pLDDT <70 were interpreted cautiously. pLDDT and PAE outputs for the wild-type and variant-specific models were retained as model-quality measures and were considered when interpreting structural differences.

Molecular docking was performed using AutoDock-Vina v.1.1.2 (Eberhardt et al., 2021). Receptor and ligand preparation was undertaken using UCSF Chimera v.1.18 (Rodríguez-Guerra Pedregal and Maréchal, 2018). Three-dimensional structures of eight opioid ligands—buprenorphine, fentanyl, methadone, morphine, naloxone, oxycodone, remifentanil, and sufentanil—were obtained from PubChem release 2024 (Kim et al., 2021). Ligand structures were converted to MOL2 format, protonated where appropriate, and energy minimised before docking.

The docking search space was centred on the established μ-opioid receptor ligand-binding pocket, based on published structural and receptor–ligand interaction studies (Cong et al., 2021; Serohijos et al., 2011; Xie et al., 2022; Ricarte et al., 2021; Zhuang et al., 2022; Zhang et al., 2022). The same docking-box coordinates and dimensions were used for the wild-type and all variant-specific receptor models to permit consistent within-ligand comparisons.

AutoDock Vina was configured to generate nine binding poses per receptor-ligand combination, with an exhaustiveness value of 8 and an energy range of Δ = 3 kcal/mol. Poses were ranked using the AutoDock Vina scoring function. Representative poses were selected according to predicted binding energy, localisation within the expected ligand-binding pocket, and consistency with previously reported receptor–ligand contact patterns.

The receptor models were treated as rigid during docking. Receptor flexibility, induced-fit effects, molecular-dynamics refinement, and ensemble docking were not modelled. Predicted docking energies and poses were therefore interpreted as approximate comparative measures rather than experimentally validated estimates of binding affinity.

For each ligand, docking results obtained with the wild-type receptor were compared with those obtained using each variant-specific receptor model. Comparisons considered the predicted docking score, orientation within the ligand-binding pocket, and predicted receptor–ligand contacts. Each ligand was assessed independently; docking scores were not compared directly across chemically distinct ligands as quantitative measures of relative pharmacological potency.

The docking workflow was not calibrated against experimentally measured binding affinities or systematically benchmarked against experimentally resolved ligand-bound μ-opioid receptor structures. Accordingly, observed differences between wild-type and variant-specific models were interpreted as exploratory predictions of variant-associated changes in docking behaviour rather than validated evidence of altered ligand affinity, receptor function, clinical drug response, or pharmacogenomic actionability.

Post-docking structural analysis was performed using UCSF ChimeraX v.1.9 (Pettersen et al., 2021). Predicted ligand–receptor interactions, hydrogen bonds, binding-pocket residues, and interatomic distances were examined for selected poses. These analyses were intended to provide structural context for selected *OPRM1* missense variants. Docking scores and predicted poses were not used to assign pharmacogenomic phenotypes, infer opioid dose requirements, or define clinical actionability. Experimental functional and pharmacological studies would be required to validate any predicted structural or ligand-binding effects.

### 2.7 Data visualisation and figure generation

Plots and figures were created using R and Python. Statistical charts were generated with ggplot2 v.3.5.1 (R; https://github.com/tidyverse/ggplot2) and Matplotlib v.3.11.1 (Python; https://github.com/matplotlib/matplotlib), and categorical relationships were visualized using ggsankey v.0.0.9 (R; https://github.com/davidsjoberg/ggsankey). Final figures were exported in raster and vector formats as appropriate for publication.

## 3 Results

### 3.1 Summary of pharmacogenetic variation

We analysed WGS data from 3,205 participants in MGRB, a healthy older Australian reference cohort enriched for individuals aged ≥70 years and predominantly of European ancestry. Accordingly, the reported prevalence estimates should be interpreted as estimates for this specific healthy older reference cohort rather than as directly generalisable estimates for all Australians or other ancestry groups.

The initial pharmacogenetic catalogue comprised 487 curated variants across 42 pharmacogenes assembled from CPIC guideline resources, ClinPGx/PharmGKB clinical annotations, and PharmVar or other star-allele reference definitions, as described in Section 2.2. Four genes—CFTR, EGFR, VKORC1, and XRCC1—were excluded *a priori* from downstream analyses, leaving 38 pharmacogenes in the VIP-wide analytic set.

ClinPGx/PharmGKB clinical annotation Levels 1A, 1B, 2A, and 2B were used to define the evidence categories represented in the VIP-wide analysis. CPIC guideline resources provided complementary prescribing, allele-function, and clinical interpretation context. Genotype distributions and allele-frequency summaries for the curated Level 1A–2B variants across the 38-gene analytic set are presented in Supplementary Table S4.

### 3.2 Prevalence and classification of high-risk level 1A configurations

Within the 38-gene analytic set, 12 pharmacogenes were prespecified as the focused high-risk subset on the basis of ClinPGx/PharmGKB Level 1A evidence, high-priority electronic health record or clinical actionability notation, and CPIC guideline-based prescribing relevance, as described in Sections 2.2–2.4.

Participant-level findings were classified using the hierarchical rsID-defined framework described in Section 2.4. Homozygous non-reference genotypes were considered phase-independent confirmed homozygous configurations. By contrast, within-gene configurations involving two or more distinct qualifying variants were not considered confirmed compound heterozygous or confirmed biallelic unless direct trans phase could be demonstrated.

Overall, 1,303 of 3,205 participants carried at least one confirmed homozygous ClinPGx/PharmGKB Level 1A high-risk configuration, corresponding to a confirmed phase-independent prevalence of 40.7% (Figure 1A). A further 409 participant–gene configurations involving 399 unique participants were classified as potential trans, phase unresolved. These 399 participants represented 12.4% of the cohort.

**FIGURE 1:**
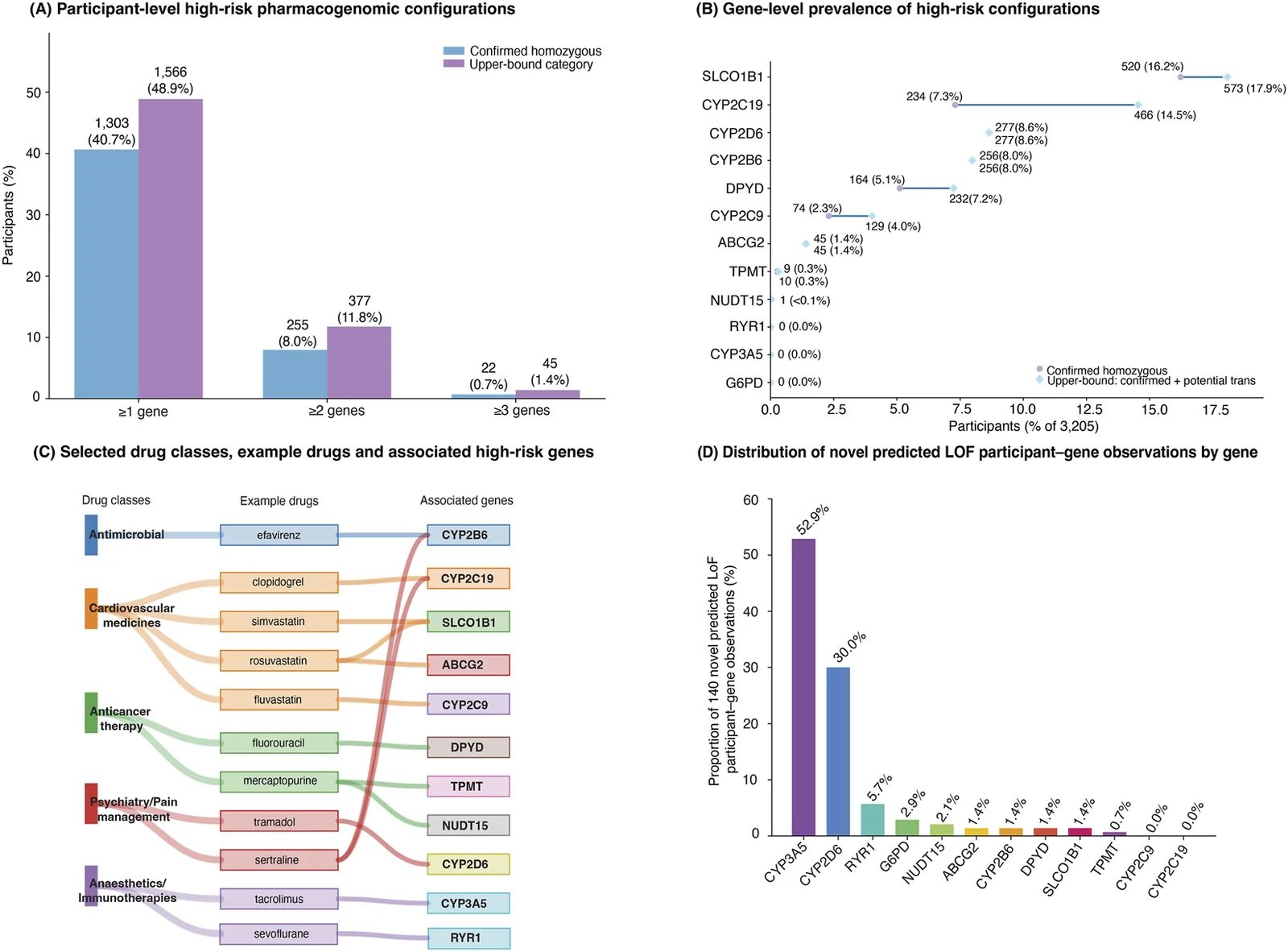
Pharmacogenomic landscape of high-risk and novel predicted loss-of-function variants in ClinPGx/PharmGKB pharmacogenes. **(A)** Participant-level prevalence of confirmed homozygous high-risk configurations and the broader upper-bound category across the 12-gene ClinPGx/PharmGKB Level 1A high-risk subset. The upper-bound category comprised participants with either a confirmed homozygous configuration or a potential-trans, phase-unresolved configuration. Values are shown for participants with configurations in at least one, at least two, and at least three high-risk pharmacogenes. The upper-bound estimates should not be interpreted as confirmed biallelic prevalence. **(B)** Paired lollipop plot showing gene-level prevalence of confirmed homozygous configurations and the corresponding upper-bound prevalence, defined as confirmed homozygous plus potential-trans, phase-unresolved configurations. Potential-trans configurations were not directly phase confirmed and therefore do not represent confirmed compound-heterozygous or confirmed biallelic findings. **(C)** Sankey diagram showing selected drug classes, example drugs, and associated high-risk pharmacogenes based on curated ClinPGx/PharmGKB and CPIC-derived annotations. The displayed drugs are illustrative examples rather than the complete scope of current prescribing recommendations for each gene. G6PD was included in the 12-gene high-risk analytic set but is not displayed in this panel. **(D)** Distribution of novel predicted loss-of-function variant observations across the high-risk pharmacogene set. Percentages represent the proportion of the 140 novel predicted loss-of-function participant–gene observations assigned to each gene and should not be interpreted as gene-specific participant prevalence, cohort prevalence, or clinical risk.

The confirmed homozygous and potential-trans groups were not mutually exclusive at the participant level. A total of 136 participants, representing 4.2% of the cohort, carried at least one confirmed homozygous configuration and at least one separate potential-trans, phase-unresolved configuration. After removing this overlap, 1,566 of 3,205 participants, or 48.9% of the cohort, had either a confirmed homozygous configuration or a potential-trans, phase-unresolved configuration. This combined value is presented as an upper-bound estimate of potential biallelic prevalence and should not be interpreted as the prevalence of confirmed compound heterozygosity or confirmed biallelic pharmacogenetic variation (Figure 1A). Multi-gene involvement was also observed and is described in Section 3.3.

The most frequently represented high-risk pharmacogenes following the revised participant-level classification were *CYP2B6*, *CYP2C19*, *CYP2D6*, *DPYD*, and *SLCO1B1* (Figure 1B). Gene-level values in Figure 1B distinguish the prevalence of confirmed homozygous configurations from the corresponding upper-bound prevalence, defined as the combined prevalence of confirmed homozygous and potential-trans, phase-unresolved configurations. Configurations classified as likely cis or haplotype-associated, as well as otherwise unresolved multi-variant configurations, were excluded from both the confirmed homozygous prevalence estimates and the upper-bound category. Because *CYP2D6* copy-number variation, gene deletions or duplications, *CYP2D6*–*CYP2D7* hybrid alleles, and other complex structural configurations were not comprehensively resolved, the *CYP2D6* values shown here represent rsID-defined configuration prevalence rather than complete *CYP2D6* diplotype or metaboliser-phenotype prevalence.

Figure 1C presents selected drug classes, example drugs, and their associated high-risk pharmacogenes based on the curated ClinPGx/PharmGKB and CPIC-derived annotations. The examples include efavirenz, clopidogrel, simvastatin, rosuvastatin, fluvastatin, fluorouracil, mercaptopurine, tramadol, sertraline, tacrolimus, and sevoflurane. These examples are illustrative and do not represent the complete scope of CPIC prescribing recommendations for each gene. For example, although simvastatin, rosuvastatin, and fluvastatin are displayed as matched statin annotations, CPIC guidance includes recommendations for multiple statins. *G6PD* was included in the 12-gene high-risk analytic set but was not displayed in Figure 1C.

Collectively, the high-risk pharmacogenes represented in Figure 1 are relevant to treatment decisions involving antimicrobial, cardiovascular, anticancer, psychiatric, analgesic, immunosuppressive, anaesthetic, and other clinically important drug classes.

At the rsID level, a relatively small number of recurrent ClinPGx/PharmGKB Level 1A configurations accounted for many of the observed within-gene multi-variant patterns before haplotype and phase refinement. Frequently observed configurations included *CYP2D6* variants involving rs1065852, rs28371703, and rs3892097; *CYP2C19* variants involving rs12769205 and rs4244285; the higher-order *CYP2C19* configuration involving rs12248560, rs12769205, and rs4244285; and *CYP2B6* variants involving rs2279343 and rs3745274. Additional recurrent observations included homozygous *CYP2C19* rs12248560, other recurrent *CYP2D6* configurations, and high-risk configurations in *DPYD* and *CYP2C9*.

These rsID-level configurations involved variants represented in established ClinPGx/PharmGKB, CPIC, or PharmVar allele definitions. However, definitive star-allele haplotypes and diplotypes were not assigned. Some recurrent multi-variant configurations were compatible with recognised same-haplotype structures rather than variants located on opposite homologues. For example, the *CYP2B6* rs3745274–rs2279343 combination was considered compatible with a CYP2B6*6*-*associated haplotype structure*.* Other recognised combinations involving CYP2C19, DPYD, SLCO1B1*15, and TPMT*3A were similarly classified as likely cis or haplotype-associated where supported by established allele definitions.

Configurations involving variants associated with distinct or potentially independent allele structures were classified as potential trans, phase unresolved when they could plausibly occur on opposite homologues, but direct participant-level phase evidence was unavailable or incomplete. These configurations were not described as confirmed compound heterozygous or confirmed trans.

To determine whether any potential-trans configurations could be resolved directly, genotype-level phase fields in the chromosome-specific MGRB VCFs were examined for all 409 candidate participant–gene configurations. The phase audit evaluated phased GT, physical phasing genotype PGT, PID, and PS information across the relevant variants.

None of the 409 candidate configurations could be directly resolved as trans or cis. Although the chromosome-specific VCF headers contained PGT, PID, and PS fields, these fields were absent or incomplete across the required variants in almost all candidate configurations. In isolated instances, phase information was available for an individual variant, but the remaining variants in the same participant–gene configuration lacked complete shared-phase-block information. Consequently, no configuration met the prespecified requirement that all relevant heterozygous variants have usable phased genotypes within the same physical phase block.

The phase audit therefore identified no directly phased trans configurations and no directly phased cis configurations among the candidate potential-trans group. All 409 participant–gene configurations remained classified as potential trans, phase unresolved.

Variant-level genotype distributions for the ClinPGx/PharmGKB Level 1A high-risk set, including heterozygous and homozygous non-reference genotypes and rsID-defined multi-variant observations, are provided in Supplementary Table S5. The haplotype and phase-interpretability review is summarised in Supplementary Table S6, while the final mutually exclusive participant–gene classifications and phase-status categories are reported in Supplementary Table S7.

### 3.3 Multi-gene high-risk pharmacogenomic configurations

Multi-gene involvement was evaluated using the final participant–gene classification framework described in Section 2.4. Among participants with confirmed phase-independent homozygous configurations, 255 of 3,205 individuals (8.0%) had qualifying configurations in two or more high-risk pharmacogenes, and 22 individuals (0.7%) had qualifying configurations in three or more genes.

A broader upper-bound analysis additionally included potential-trans, phase-unresolved configurations. Under this definition, 377 of 3,205 participants (11.8%) had either confirmed homozygous or potential-trans, phase-unresolved configurations in two or more high-risk pharmacogenes, and 45 participants (1.4%) had such configurations in three or more genes. Because the potential-trans configurations were not directly phase confirmed, these values represent upper-bound estimates of multi-gene potential biallelic involvement rather than confirmed multi-gene biallelic prevalence.

Potential-trans, phase-unresolved configurations alone were observed in two or more genes in 10 participants (0.3%); no participant had potential-trans, phase-unresolved configurations in three or more genes. These findings demonstrate that most multi-gene involvement in the upper-bound analysis was driven by confirmed homozygous configurations or by combinations of confirmed homozygous and unresolved potential-trans findings, rather than by multiple potential-trans configurations alone.

Multi-gene configurations occurred across several clinically relevant prescribing contexts. For statin-associated pharmacogenes, recurrent patterns involved *SLCO1B1* together with *CYP2C9* or *ABCG2*, which encode proteins involved in statin transport, disposition, and susceptibility to statin-associated adverse effects. The clinical relevance of these combinations varies according to the individual statin, variant context, and applicable prescribing guideline (Cooper-DeHoff et al., 2022; Figure 1C).

Within psychiatric and neuropharmacological prescribing contexts, recurrent multi-gene patterns involved *CYP2C19* and *CYP2D6*, which contribute to the metabolism of several antidepressants and other psychotropic medicines. Additional patterns involved *CYP2B6* and *CYP2C19*, genes relevant to selected antidepressant and antiretroviral prescribing contexts (Austin-Zimmerman et al., 2021; Yuce-Artun et al., 2016).

Within anticancer and immunosuppressive treatment contexts, multi-gene patterns involved *DPYD*, *NUDT15*, and *TPMT*. These genes are relevant to fluoropyrimidine- and thiopurine-associated toxicity, although their clinical effects are drug specific and cannot be inferred from genotype prevalence alone (Figure 1C).

These observations illustrate the population-level occurrence of pharmacogenomic findings across more than one clinically relevant gene. However, concurrent medication exposure, prescribing indication, dose, comorbidity, and clinical outcomes were not uniformly available for the MGRB cohort. The observed patterns should therefore be interpreted as genomic configuration profiles and not as evidence that participants were exposed to the corresponding medicines or experienced altered efficacy, toxicity, or adverse drug reactions.

The confirmed and upper-bound multi-gene estimates were reported separately to preserve the distinction between phase-independent homozygous findings and unconfirmed potential-trans configurations. Likely cis or haplotype-associated configurations and otherwise unresolved multi-variant configurations were not included in either the confirmed homozygous count or the potential-trans upper-bound category. Final participant-gene classifications supporting this analysis are provided in Supplementary Table S7.

### 3.4 Novel predicted loss-of-function variants in high-risk pharmacogenes

Most clinical pharmacogenomic assays target predefined, previously characterised alleles and may therefore fail to detect rare or previously uncatalogued variants. Using the criteria defined in Section 2.5, 140 novel predicted loss-of-function participant–gene category records were identified across the high-risk pharmacogene set. These comprised 132 heterozygous, one homozygous, and seven unresolved within-gene multi-variant findings. Detailed variant-level and participant–gene classifications are provided in Supplementary Table S10. No novel predicted loss-of-function variants were identified in *CYP2C9* or *CYP2C19*.

Eight participant–gene records were classified as either homozygous or unresolved within-gene multi-variant configurations. Homozygous novel variants were considered phase independent. In contrast, configurations involving two or more distinct novel variants within the same gene were not classified as confirmed compound heterozygous, confirmed trans, or confirmed biallelic findings in the absence of direct phase evidence. Novel multi-variant configurations lacking sufficient participant-level phase evidence were retained as unresolved findings requiring further validation.

The distribution of novel predicted loss-of-function observations across the high-risk pharmacogenes is shown in Figure 1D. These percentages describe the distribution of novel observations across genes and do not represent gene-specific participant prevalence, clinical risk, altered drug response, or confirmed pharmacogenomic actionability.

These findings illustrate the additional discovery capacity of whole-genome sequencing compared with assays restricted to established pharmacogenetic alleles. Novel variants were not included in the primary ClinPGx/PharmGKB Level 1A prevalence estimates unless they independently overlapped with established clinical evidence. Predicted loss of function alone was not considered sufficient evidence of clinical actionability. The identified variants should therefore be regarded as putatively impactful candidates requiring gene-specific functional assessment, population-frequency evaluation, clinical correlation, and, where relevant, phase-resolved validation before clinical interpretation.

### 3.5 Pharmacogenomic landscape of known and candidate variants in opioid-response pathways

Opioid pharmacogenetics was selected for detailed exploratory evaluation because of the clinical importance of opioid analgesics and the availability of structural and functional information for genes involved in opioid metabolism and signalling (Crist and Berrettini, 2014). The analysis included pharmacogenes involved in opioid disposition as well as opioid receptor genes. These exploratory findings were evaluated separately from the primary ClinPGx/PharmGKB Level 1A high-risk prevalence analysis.

Among the metabolic pharmacogenes, CYP2D6 was of particular interest because of its established role in the bioactivation or metabolism of several opioid medicines, including codeine and tramadol. Using the final participant–gene classification framework described in Section 2.4, 277 of 3,205 participants (8.6%) carried a confirmed homozygous ClinPGx/PharmGKB Level 1A *CYP2D6* configuration. No *CYP2D6* configurations were classified as potential trans, phase unresolved. Consequently, the *CYP2D6* upper-bound estimate was also 277 participants (8.6%).

An additional 658 participants had *CYP2D6* multi-variant configurations classified as likely cis or haplotype-associated, while 426 had unresolved multi-variant configurations. These findings were excluded from both the confirmed homozygous prevalence and the upper-bound estimate. Because *CYP2D6* is structurally complex, comprehensive functional interpretation requires copy-number-aware, hybrid-allele-aware, and phase-resolved analysis. The present rsID-defined short-read WGS framework did not comprehensively resolve *CYP2D6* whole-gene deletions such as CYP2D6*5, gene duplications, *CYP2D6*–*CYP2D7* hybrid alleles, or other complex structural configurations. Accordingly, the observed *CYP2D6* findings were not interpreted as definitive diplotypes, activity scores, or metaboliser phenotypes, and the reported *CYP2D6* prevalence estimates should not be interpreted as the prevalence of functional *CYP2D6* metaboliser phenotypes in the cohort. The final *CYP2D6* participant-level classifications are provided in Supplementary Table S7.

Candidate *CYP2D6* findings outside the primary curated Level 1A classification were uncommon and were retained separately as exploratory observations. These variants were not assigned definitive *CYP2D6* haplotypes or functional phenotypes. No novel predicted loss-of-function variants were identified in *CYP2C19*. Although *CYP2C19* has been investigated in selected opioid-related pharmacogenomic contexts, including oxycodone disposition, *CYP2C19* findings were interpreted as part of the broader pharmacogenomic landscape rather than as definitive predictors of opioid response in this cohort (Zhu et al., 2022).

Opioid receptor genes were also examined, including *OPRM1*, which encodes the μ-opioid receptor; *OPRK1*, which encodes the κ-opioid receptor; and *OPRL1*, which encodes the nociceptin receptor (Machelska and Celik, 2020). These receptor genes did not contain ClinPGx/PharmGKB Level 1A–2B annotations in the curated variant dataset and were therefore not included in the primary clinically actionable high-risk analysis.

A total of 26 *OPRM1* variants were identified in the MGRB cohort, including four missense variants and additional synonymous, intronic, and 3′untranslated-region variants. Three participants carried rare heterozygous stop-gain variants, and ten carried rare heterozygous missense variants classified as candidate findings under the criteria described in Section 2.5. Protein-truncating variants, including stop-gain and frameshift variants, were regarded as predicted loss-of-function candidates but were not assumed to confer pharmacogenomic actionability.

These findings demonstrate additional variation in genes involved in opioid-response pathways that may not be represented in standard allele-targeted pharmacogenomic assays. However, the functional and clinical consequences of these variants remain uncertain. Candidate opioid-pathway variants were therefore retained as exploratory observations and were not used to define the primary ClinPGx/PharmGKB Level 1A high-risk prevalence estimates.

### 3.6 Exploratory structural modelling and molecular docking of *OPRM1* missense variants

To explore the potential structural effects of selected *OPRM1* missense variants, *in silico* receptor modelling and molecular docking were performed using the OPRM1-201 transcript model, ENST00000229768.9, as the reference sequence, as described in Section 2.6. This analysis was exploratory and was conducted separately from the primary ClinPGx/PharmGKB Level 1A analysis. Model confidence was evaluated using AlphaFold-derived predicted local distance difference test scores and predicted aligned-error matrices, and structural interpretations were restricted to receptor regions with acceptable local confidence.

Variants were prioritised for modelling when they represented coding missense substitutions that could be mapped unambiguously to the selected *OPRM1* protein model and had sufficient cohort or annotation information to support exploratory comparison. Models were generated for rs77013544 and rs1799974, observed in approximately 0.06% and 2.0% of MGRB participants, respectively, together with four ClinPGx/PharmGKB Level 3 variants: rs1799971, rs79910351, rs62638690, and rs540825, observed in approximately 22.0%, 5.6%, 1.4%, and 93.0% of participants, respectively. These percentages represent participant carrier frequencies rather than cohort allele frequencies.

In this study, rs77013544 and rs1799974 were considered novel only with respect to their absence from the predefined curated pharmacogenomic catalogue, where applicable; this designation does not imply that the variants had not previously been reported. The four Level 3 variants were included as exploratory structural comparators because they had lower-level pharmacogenomic annotations or previously reported associations with opioid-related phenotypes. They were not considered clinically actionable high-risk variants and were not included in the Level 1A prevalence estimates.

ClinPGx/PharmGKB Level 3 annotations do not provide the same strength of clinical evidence as Level 1A annotations. Nevertheless, several of the modelled variants have previously been investigated in relation to opioid-associated phenotypes. These include rs79910351 in studies of opioid response, rs62638690 in addiction-related phenotypes, rs540825 in fentanyl-associated emesis, and rs1799971, commonly described as A^118^G, in a range of opioid-response studies (Clarke et al., 2013; Pang et al., 2012; Hajj et al., 2017; Skorpen et al., 2016; Ols Olsen et al., 2019). These published associations were used only to provide context for exploratory modelling and were not interpreted as evidence of clinical actionability in the present cohort.

Docking simulations were conducted for eight opioid ligands: buprenorphine, fentanyl, methadone, morphine, naloxone, oxycodone, remifentanil, and sufentanil. For each ligand, docking results obtained with the wild-type receptor model were compared with results obtained using each variant-specific receptor model. Comparisons were therefore performed within each ligand, such as wild-type *OPRM1*–fentanyl compared with variant *OPRM1*–fentanyl, rather than across chemically distinct ligands.

Among the modelled receptor–ligand combinations, fentanyl showed the largest variation in predicted docking score across the *OPRM1* models. The wild-type receptor model produced a predicted fentanyl docking score of −6.23 kcal/mol, whereas the rs79910351 model produced a lower score of −8.39 kcal/mol. Within the assumptions of the docking model, the lower score was consistent with a more favourable predicted docking pose. Other ligands showed smaller differences across the modelled receptor variants (Table 1; Supplementary Figure S2).

**TABLE 1:** Change in predicted binding energy relative to wild-type *OPRM1* for selected opioid ligands docked to *OPRM1* variant receptor models.

Parameter/ligand	rs540825	rs1799971	rs62638690	rs79910351	rs1799974	rs77013544
Population allele frequency	74.4%	12.4%	0.73%	0.28%	0.12%	0.03%
Amino-acid substitution	Gln^402^His	Asn^40^Asp	Cys^192^Phe	Arg^181^Cys	Arg^260^His	Arg^347^Gln
Nucleotide substitution	A>T (1,256)	A>G (168)	G>T (625)	C>T (591)	G>A (892)	G>A (1,090)
Δ binding energy vs. WT (kcal/mol)						
Buprenorphine	0.29	0.49	0.05	−0.01	0.16	0.20
Fentanyl	−1.89	−1.83	−1.92	−2.16	−2.00	−2.06
Methadone	−0.49	−0.82	0.12	−0.57	−0.53	−0.72
Morphine	−0.11	−0.07	−0.11	−0.44	0.00	−0.03
Naloxone	−0.25	−0.06	−0.20	−0.60	−0.13	−0.25
Oxycodone	0.00	−0.02	0.01	−0.47	−0.08	0.09
Remifentanil	−0.06	0.13	−0.02	−0.71	0.14	−0.14
Sufentanil	−0.06	−0.06	−0.70	0.09	−0.06	−0.02

WT, wild type; Δ, change relative to the wild-type *OPRM1* receptor model. Negative Δ values indicate lower predicted binding energy relative to WT, in the docking model. Population allele frequencies are allele-level frequencies and are not equivalent to the observed MGRB, carrier frequencies reported in the Results text.

These results provide exploratory structural context only. The predicted docking scores were not experimentally calibrated and should not be interpreted as measured binding affinities, receptor activation, opioid dose requirements, adverse-effect risks, or clinical pharmacogenomic phenotypes.

### 3.7 Predicted differences in fentanyl–OPRM1 receptor interactions associated with rs79910351

To examine the predicted structural effect of rs79910351 on fentanyl binding, the wild-type *OPRM1* receptor model was compared with the variant model containing the Arg^181^Cys substitution. The comparison was performed using the same ligand and docking procedure for both receptor models. Labels H^7^, H^10^, and H^15^ refer to atom or contact labels assigned to fentanyl in the docking output and do not represent receptor helices or amino-acid residue positions.

In the selected wild-type docking pose, fentanyl was positioned near Tyr328, Gln126, and Asp149, with predicted interatomic distances of 6.4^Å^, 4.0^Å^, and 5.9^Å^, respectively. Arg^181^ was located within the broader predicted binding region but did not form a close direct interaction with fentanyl in the displayed pose (Figure 2A). Because several of these distances exceed those normally expected for conventional hydrogen bonding, they are described here as predicted proximity or contact distances rather than confirmed hydrogen bonds.

**FIGURE 2:**
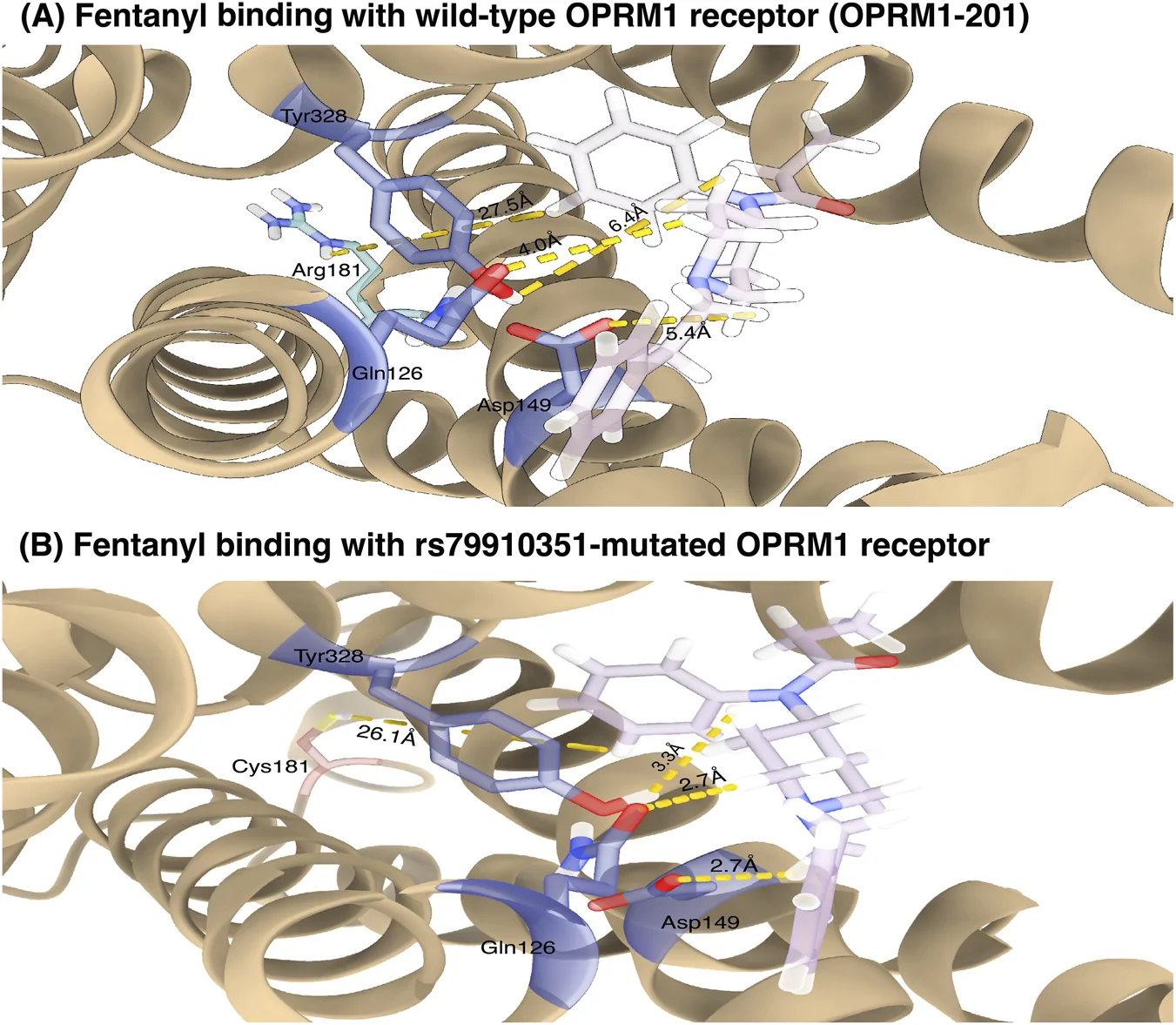
Comparative docking analysis of predicted fentanyl interactions with wild-type and rs79910351-mutant *OPRM1* receptor models. **(A)** Structural docking model showing fentanyl positioned within the wild-type μ-opioid receptor isoform OPRM1-201 (ENST00000229768.9). In the selected pose, fentanyl was located near Tyr^328^, Gln^126^, and Asp^149^, with predicted interatomic distances of 6.4^Å^, 4.0^Å^, and 5.9^Å^, respectively. Arg^181^ was located within the broader predicted binding region but did not form a close direct interaction with fentanyl in the displayed pose. Because several of these distances exceed those typically associated with conventional hydrogen bonding, they are described as predicted proximity or contact distances rather than confirmed hydrogen bonds. **(B)** Structural docking model showing fentanyl positioned within the rs79910351-mutant receptor model, corresponding to the Arg^181^Cys amino-acid substitution. The mutant model showed altered predicted ligand orientation and local contact geometry, including predicted distances of 3.3^Å^ between the H^7^-labelled fentanyl atom and Tyr^328^, 2.7^Å^ between the H^10^-labelled atom and Cys^181^, and 2.7^Å^ between the H^15^-labelled atom and Asp^149^. H^7^, H^10^, and H^15^ denote labelled fentanyl atom or contact positions in the docking output and do not refer to receptor helices or amino-acid residue numbers. These findings provide exploratory structural context only and should not be interpreted as validated evidence of altered ligand affinity, receptor activation, opioid response, dose requirement, adverse-effect risk, or clinical pharmacogenomic actionability. Experimental validation is required to determine whether the predicted differences have functional consequences.

In the selected rs79910351 model, replacement of Arg^181^ with cysteine was associated with altered predicted ligand orientation and local contact geometry. The distance between the fentanyl H^7^-labelled atom and Tyr328 was reduced to 3.3Å, while H10- and H15-labelled atoms showed predicted distances of approximately 2.7^Å^ from Cys181 and Asp149, respectively (Figure 2B). These contacts were highlighted because they represented the clearest differences between the selected wild-type and variant docking poses.

The altered predicted contact geometry and lower fentanyl docking score observed for the rs79910351 model suggest that the Arg^181^Cys substitution may influence the positioning of fentanyl within the modelled receptor-binding pocket. However, this interpretation is limited to the selected static docking poses. The analysis did not model receptor flexibility, induced fit, solvent effects, membrane context, or molecular dynamics and was not benchmarked systematically against experimentally resolved fentanyl-bound μ-opioid receptor structures.

Accordingly, these findings should be interpreted as hypothesis-generating structural predictions rather than evidence of altered ligand affinity, receptor activation, opioid response, dose requirement, adverse-effect risk, or clinical actionability. Experimental receptor-binding, signalling, and pharmacological studies would be required to determine whether the predicted structural differences have functional consequences.

## 4 Discussion

This study characterised clinically relevant pharmacogenomic variation in 3,205 participants from a healthy older Australian reference cohort using a focused, phase-aware framework. Across 12 high-risk pharmacogenes selected on the basis of ClinPGx/PharmGKB Level 1A evidence and CPIC guideline-based prescribing relevance, 1,303 participants (40.7%) carried at least one confirmed homozygous non-reference configuration. An additional 399 participants (12.4%) carried at least one potential-trans, phase-unresolved configuration. Because 136 participants occurred in both groups, the nonduplicated combined category included 1,566 participants (48.9%). This value represents an upper-bound estimate of potential biallelic involvement and should not be interpreted as confirmed biallelic prevalence.

Multi-gene involvement was similarly dependent on the classification applied. Confirmed homozygous configurations were present in two or more high-risk pharmacogenes in 255 participants (8.0%) and in three or more genes in 22 participants (0.7%). When potential-trans, phase-unresolved configurations were additionally included, the corresponding upper-bound estimates increased to 377 participants (11.8%) with findings in two or more genes and 45 participants (1.4%) with findings in three or more genes. Reporting these categories separately preserves the distinction between phase-independent homozygous findings and multi-variant configurations for which trans orientation could not be demonstrated.

The prevalence estimates reported here are lower than broader estimates of pharmacogenomic actionability reported in other studies. Previous analyses have often included heterozygous carriers, lower evidence tiers, larger gene panels, copy-number variation, classical *HLA* alleles, or phenotype-based diplotype inference (Swen et al., 2023; Verma et al., 2022; Hodel et al., 2024; Chanfreau-Coffinier et al., 2019; Lunenburg et al., 2021). In contrast, the primary analysis in this study was restricted to rsID-defined ClinPGx/PharmGKB Level 1A variants in a predefined high-risk gene set. Definitive star alleles, diplotypes, metaboliser phenotypes, participant-level activity scores, *CYP2D6* copy-number or hybrid alleles, and classical *HLA* alleles were not assigned.

Classical pharmacogenomic *HLA* alleles, including *HLA*-B*57:01, *HLA*-B*15:02, *HLA*-B*58:01, and *HLA*-A*31:01, were excluded because validated *HLA* typing or imputation was outside the scope of the rsID-defined SNV and short-indel workflow. Their exclusion reflects the analytic boundaries of the study and should not be interpreted as indicating limited clinical importance. The resulting estimates therefore describe high-priority pharmacogenomic variation detectable within the limits of the present variant-centric WGS pipeline rather than the complete pharmacogenomic actionability of the cohort.

Homozygous and confirmed trans pharmacogenetic configurations may have greater functional consequences than isolated heterozygous variants in several clinically important pharmacogenes. Relevant examples include *TPMT* and *NUDT15* variation in thiopurine sensitivity (Sanderson, 2015; Yu et al., 2020; Yu et al., 2023), *DPYD* variation in fluoropyrimidine toxicity risk (de Haar-Holleman et al., 2024), and *RYR1* variation in malignant-hyperthermia susceptibility (Janßen et al., 2024; Kraeva et al., 2015). In the present cohort, confirmed homozygous configurations were observed across multiple high-risk pharmacogenes. Within-gene multi-variant configurations were also common, but their interpretation required particular caution because the presence of two variants in the same gene does not establish that they occur on opposite homologues.

To reduce overinterpretation, recurrent rsID-defined multi-variant configurations were reviewed using established PharmVar, CPIC, and ClinPGx allele or haplotype definitions where available. Several recurrent configurations involving *CYP2B6*, *CYP2C19*, *DPYD*, *SLCO1B1*, and *TPMT* were compatible with recognised same-haplotype structures and were therefore classified as likely cis or haplotype associated. Other configurations were considered potentially compatible with variants on opposite homologues but were retained as potential trans, phase unresolved unless direct participant-level phase evidence was available. These interpretive categories provide useful context but do not constitute definitive star-allele, diplotype, or participant-level phase assignments.

Available genotype-level phase information was subsequently audited for all 409 participant–gene configurations classified as potential trans, phase unresolved. The audit examined GT, PGT, PID, and PS fields and required all relevant heterozygous variants to have usable phased genotypes within a shared physical phase block. None of the 409 configurations could be directly resolved as trans or cis. Although isolated variants occasionally contained phase information, no complete candidate configuration had sufficient shared-block evidence across all contributing variants. Consequently, all 409 configurations remained potential trans, phase unresolved, and none was included as a confirmed compound-heterozygous configuration.

These findings demonstrate that unphased multi-variant observations should not be incorporated into a confirmed biallelic prevalence estimate. The confirmed 40.7% prevalence therefore comprises phase-independent homozygous findings only, whereas the 48.9% combined estimate is explicitly presented as an upper bound that additionally includes unresolved potential-trans configurations. Read-backed phasing with sufficient spanning information, long-read sequencing, family-based segregation, or validated pharmacogene-specific haplotyping would be required to resolve these configurations more definitively.

Multi-gene high-risk configurations were observed across several clinically relevant prescribing contexts, including statins, psychotropic medicines, anticancer agents, and immunosuppressants. Recurrent patterns involved *SLCO1B1* with *CYP2C9* or *ABCG2* in statin-related contexts, *CYP2D6* with *CYP2C19* in selected psychiatric prescribing contexts, and *DPYD*, *TPMT*, or *NUDT15* in fluoropyrimidine- and thiopurine-related contexts. These patterns illustrate the potential value of evaluating multiple pharmacogenes simultaneously rather than interpreting individual genes in isolation. However, the clinical relevance of a multi-gene profile is drug specific and depends on the variants involved, their functional effects, medication exposure, dose, indication, comorbidities, and other clinical factors.

Medication exposure, prescribing indication, treatment dose, and clinical outcome data were not uniformly available for the MGRB cohort. The multi-gene findings should therefore be interpreted as population-level genomic configuration patterns rather than evidence that participants were exposed to the relevant medicines or experienced altered efficacy, toxicity, or adverse drug reactions. The analysis does not establish cumulative or synergistic clinical effects across pharmacogenes.

Whole-genome sequencing also identified rare and previously uncatalogued variation that would not necessarily be detected by assays restricted to established pharmacogenetic alleles. A total of 140 novel predicted loss-of-function participant–gene category records were identified across the high-risk pharmacogene set, comprising 132 heterozygous, one homozygous, and seven unresolved within-gene multi-variant findings. These observations occurred across genes including *CYP3A5*, *RYR1*, G6PD*, NUDT15*, *CYP2D6*, *TPMT*, *ABCG2*, *DPYD*, and *SLCO1B1*. No novel predicted loss-of-function variants were identified in *CYP2C9* or *CYP2C19*.

The novel findings demonstrate the additional discovery capacity of WGS, but predicted loss of function alone does not establish pharmacogenomic actionability. The identified variants were not incorporated into the primary ClinPGx/PharmGKB Level 1A prevalence estimates unless they independently overlapped with established clinical evidence. Novel homozygous findings were considered phase independent, whereas novel within-gene multi-variant configurations were retained as unresolved unless direct phase evidence was available. These variants should therefore be regarded as putatively impactful candidates requiring population-frequency assessment, gene-specific functional evaluation, clinical correlation, and, where relevant, phase-resolved validation.

As an exploratory extension, selected *OPRM1* missense variants were assessed using structural modelling and molecular docking. For each opioid ligand, predicted docking results from the wild-type receptor model were compared with those from variant-specific receptor models. The rs79910351 variant, corresponding to Arg^181^Cys, produced a lower predicted fentanyl docking score and altered local contact geometry relative to the wild-type model. These observations suggest that the substitution may influence fentanyl positioning within the modelled receptor-binding pocket.

The structural results should nevertheless be interpreted cautiously. The docking analysis used static receptor models and did not account for receptor flexibility, induced fit, solvent effects, membrane context, or molecular dynamics. It was not systematically benchmarked against experimentally resolved fentanyl-bound μ-opioid receptor structures, and the predicted docking scores were not calibrated against measured binding affinities. The findings therefore provide hypothesis-generating structural context rather than evidence of altered receptor activation, opioid dose requirement, treatment response, adverse-effect risk, or clinical pharmacogenomic actionability.

Several additional limitations should be considered. The MGRB is a healthy older Australian reference cohort enriched for participants aged 70 years or older and predominantly of European ancestry. The findings should not be interpreted as representative of all Australians or of global populations. Pharmacogene allele frequencies, haplotype distributions, and clinically actionable phenotypes vary substantially across ancestry groups (Wang et al., 2025), and selection of healthy older participants may introduce survivor and ascertainment biases.

The analysis was restricted primarily to SNVs and short indels within a variant-centric framework. *CYP2D6* copy-number variation, whole-gene deletions such as CYP2D6*5, gene duplications, *CYP2D6*–*CYP2D7* hybrid alleles, and other complex structural configurations were not comprehensively resolved. This limitation is particularly important for *CYP2D6* because accurate functional interpretation requires copy-number-aware, hybrid-allele-aware, and phase-resolved haplotyping. Accordingly, the *CYP2D6* prevalence estimates reported in this study represent rsID-defined configuration prevalence and should not be interpreted as a complete estimate of *CYP2D6* diplotypes, activity scores, or poor, intermediate, normal, rapid, or ultrarapid metaboliser phenotypes.

The direct VCF phase audit was limited by the completeness of existing phase annotations. The absence of complete shared-block phase information does not demonstrate that the candidate configurations are cis or trans; it indicates only that their orientation could not be established from the available data. Population-level linkage disequilibrium or correspondence with known star-allele structures can assist interpretation but cannot substitute for participant-level phasing. Future implementation of complementary sequencing and pharmacogene-specific analytical approaches could address several of these limitations. Read-backed phasing with sufficient spanning information and long-read sequencing could improve direct resolution of cis/trans orientation and provide extended haplotype information across complex pharmacogene loci. Long-read and structural-variant-aware approaches could also improve detection of gene deletions, duplications, and hybrid alleles, particularly at *CYP2D6*. Dedicated *HLA* typing or validated *HLA* imputation would additionally enable detection of clinically actionable classical *HLA* alleles, including *HLA*-B*57:01, *HLA-*B*15:02, *HLA*-B*58:01, and *HLA-*A*31:01, which were outside the scope of the present rsID-defined SNV and short-indel workflow. Together, these approaches would support more complete haplotype-, diplotype-, structural-variant-, and phenotype-aware pharmacogenomic interpretation.

Finally, the evidence resources used for curation represented archived 2022 releases accessed in August 2024. ClinPGx/PharmGKB, CPIC, and PharmVar annotations continue to evolve, and future reinterpretation may alter the clinical classification of individual variants or gene–drug relationships. The novel-variant and *OPRM1* modelling analyses also remain exploratory and require functional and clinical validation.

In summary, population-scale WGS can expand pharmacogenomic characterisation by identifying established and candidate variants and by describing within-gene and multi-gene configurations across clinically relevant pharmacogenes. In this healthy older Australian reference cohort, 40.7% of participants carried at least one confirmed homozygous high-risk Level 1A configuration. A broader upper-bound category that additionally included potential-trans, phase-unresolved findings involved 48.9% of participants, but should not be interpreted as confirmed biallelic prevalence. These findings emphasise both the potential value of WGS-based pharmacogenomic screening and the need for validated phasing, structural-variant detection, *HLA* typing, medication-exposure data, and clinical decision-support systems before comprehensive clinical implementation.

## Data Availability

This study used controlled-access whole-genome sequencing data from the Medical Genome Reference Bank (MGRB). Summary variant frequency data for the MGRB cohort are available through the Garvan Institute web portal. Raw MGRB genomic data have been deposited at the European Genome-Phenome Archive under study accession EGAS00001003511. Phenotype data are available upon application to the MGRB Data Access Committee at mgrb@garvan.org.au and are subject to applicable data-access approvals, governance requirements, and material transfer agreements. Individual-level genomic and phenotype data are not publicly released because of participant privacy and re-identification risk. The processed pharmacogenomic summary outputs and code generated for the present study are available from the corresponding author upon reasonable request, subject to institutional approval and data-sharing restrictions.
