# Supplementary material for: Population prevalence of high-risk pharmacogenetic configurations in a healthy older Australian reference cohort
Source: Front Pharmacol. 2026 Sep 17;17:1881869. doi: 10.3389/fphar.2026.1881869 (PMC13627787; doi:10.3389/fphar.2026.1881869)
Supplement: Supplementary file 1 [file DataSheet1.pdf]

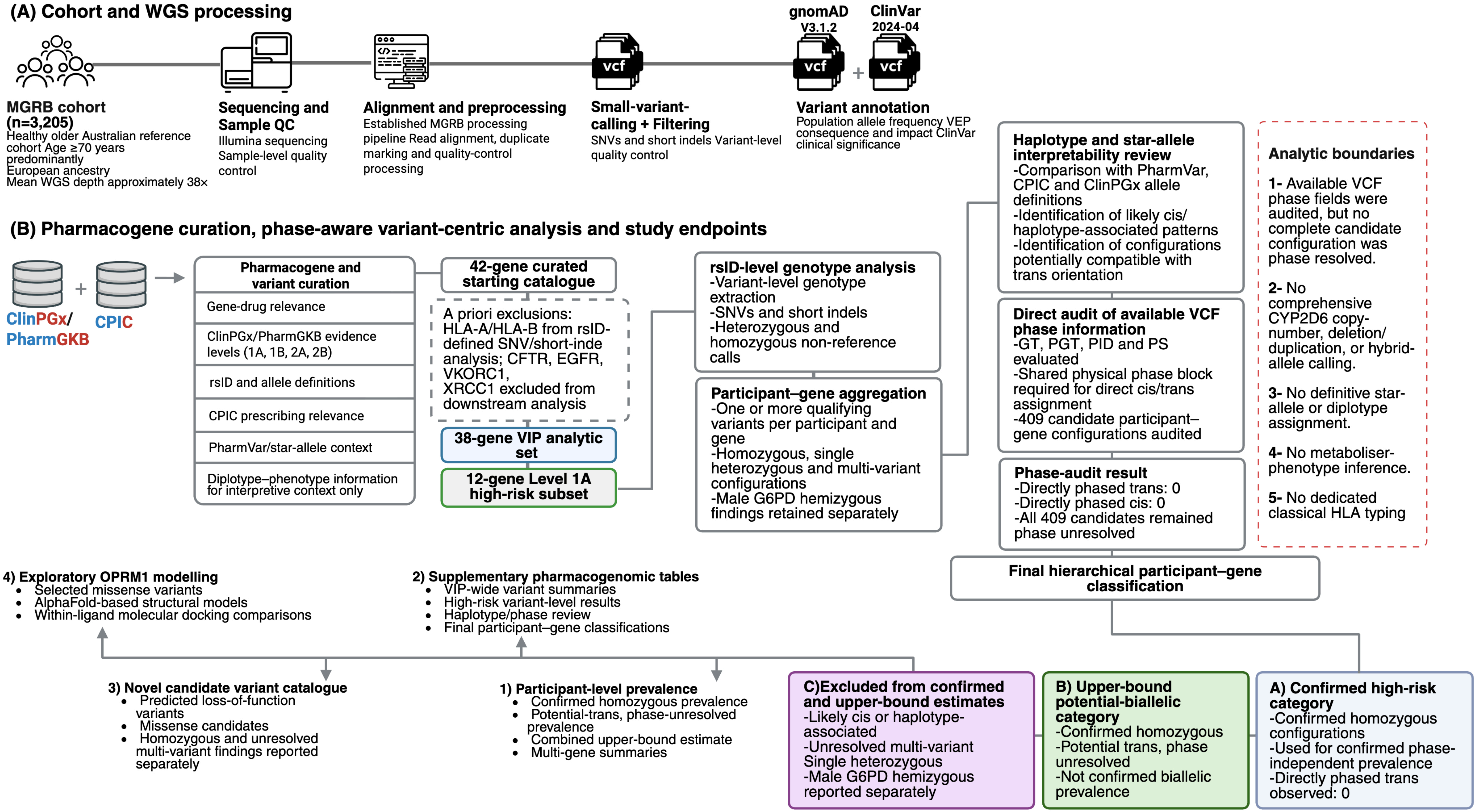

Figure S1. **Overview of study design and analysis workflow.(A)** Whole-genome sequencing data from 3,205 participants in the Medical Genome Reference Bank were processed using the established MGRB pipeline, aligned to GRCh38, subjected to small-variant calling and quality control, and annotated using population-frequency, consequence, and clinical-interpretation resources. **(B)** Pharmacogenes and variants were curated from archived ClinPGx/PharmGKB and CPIC resources, producing an initial 42-gene catalogue. After predefined exclusions, 38 genes were retained for the VIP-wide analysis and 12 genes for the focused high-risk analysis. Genotypes were analysed at the rsID level and aggregated by participant and gene. Recurrent within-gene multi-variant configurations were reviewed using established PharmVar, CPIC, and ClinPGx allele or haplotype definitions. Available VCF phase fields (GT, PGT, PID, and PS) were then audited for configurations considered potentially compatible with trans orientation. Final participant–gene classifications were assigned hierarchically as confirmed homozygous, directly phased trans, potential trans with phase unresolved, likely cis or haplotype-associated, unresolved multi-variant, or single heterozygous; male G6PD hemizygous findings were retained separately. No candidate configuration was directly resolved as trans or cis. Confirmed homozygous findings were used for the phase-independent prevalence estimate, whereas potential-trans, phase-unresolved findings were included only in an explicitly labelled upper-bound estimate. Definitive star alleles, diplotypes, metaboliser phenotypes, CYP2D6 copy-number or hybrid alleles, and classical HLA alleles were not assigned. Final outputs included cohort prevalence summaries, variant-level and participant-level supplementary tables, a novel predicted loss-of-function candidate catalogue, and exploratory OPRM1 structural modelling and molecular docking.
