# Supplementary material for: Population prevalence of high-risk pharmacogenetic configurations in a healthy older Australian reference cohort
Source: Front Pharmacol. 2026 Sep 17;17:1881869. doi: 10.3389/fphar.2026.1881869 (PMC13627787; doi:10.3389/fphar.2026.1881869)
Supplement: Supplementary file 2 [file DataSheet2.pdf]

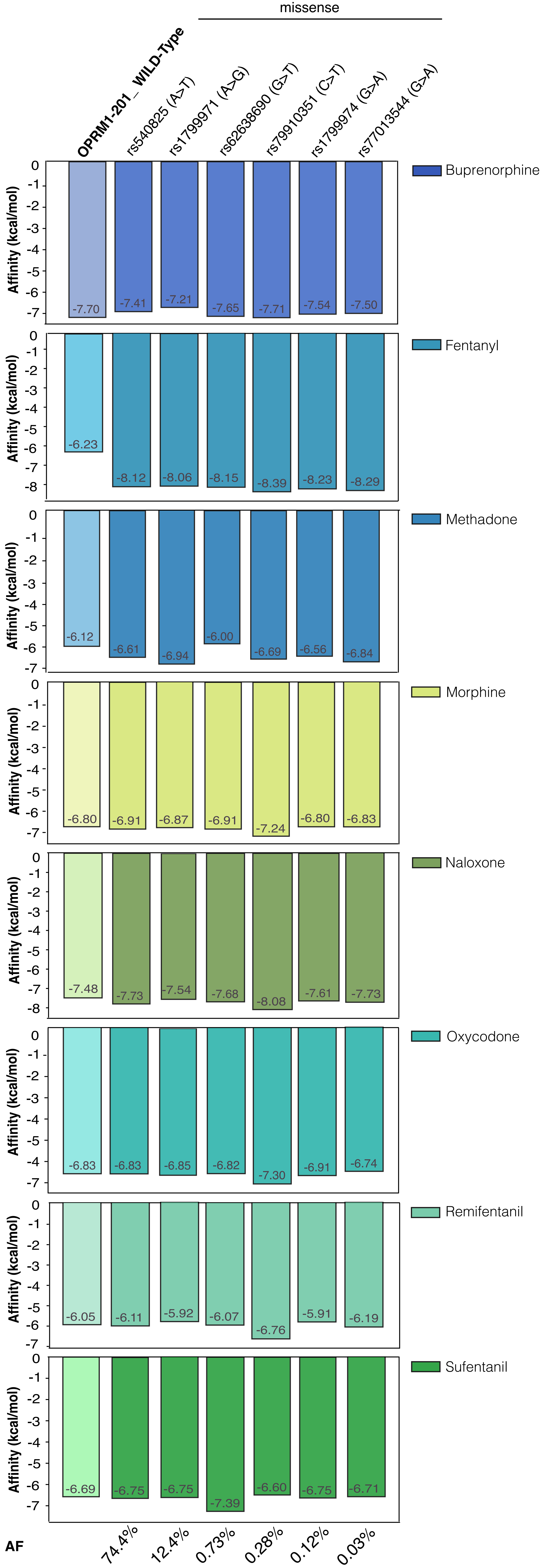

Figure S2. **Binding Affinity Comparison of Opioid Drugs with Wild-Type and Mutant OPRM1 Variants.** Bar plots illustrate the predicted binding affinities (in kcal/mol) of eight opioids—buprenorphine (MediumSlateBlue), fentanyl (DeepSkyBlue3), methadone (DodgerBlue3), morphine (DarkOliveGreen1), naloxone (OliveDrab4), oxycodone (MediumTurquoise), remifentanil (Aquamarine), and sufentanil (SpringGreen)—to the wild-type μ-opioid receptor (OPRM1-201) and six modeled missense variants: rs77013544, rs1799974, rs1799971, rs79910351, rs62638690, and rs540825. Molecular docking simulations were conducted to evaluate changes in receptor–ligand interactions caused by these variants.
